# Community Co‐Design of a Quick Guide to Inform Digital Health Promotion Strategies for Asymptomatic Women at Risk of Chronic Disease

**DOI:** 10.1002/hpja.70222

**Published:** 2026-07-26

**Authors:** Paola Borquez‐Arce, Eva Ignatious, Kym Berchtenbreiter, Jazmina L. Gonzalez Cruz, Jacqueline Frayne, Jennifer Stone

**Affiliations:** ^1^ School of Population and Global Health The University of Western Australia Perth Western Australia Australia; ^2^ Faculty of Science and Technology Charles Darwin University Darwin Northern Territory Australia; ^3^ The University of Western Australia Australian Breast Density Consumer Advisory Council Perth Western Australia Australia; ^4^ Frazer Institute The University of Queensland Brisbane Queensland Australia; ^5^ Medical School, General Practice The University of Western Australia Perth Western Australia Australia

**Keywords:** chronic disease risk management, co‐design methodology, digital health promotion, women's health

## Abstract

**Introduction:**

Chronic diseases are a leading cause of illness, disability, and death in Australian women. Co‐design research methodologies offer promising approaches to developing community‐centred solutions that improve engagement with preventive health. This community‐driven research explored ways to improve the prevention and early detection of chronic diseases in healthy women (without symptoms of disease).

**Methods:**

A Double Diamond approach guided the project. Areas of interest were identified through a Community Conversation, followed by co‐investigation of digital health promotion strategies for women. An online survey (*n* = 145) and in‐depth interviews (*n* = 21) explored women's knowledge of chronic disease risk, risk prediction tools and screening. Thematic analysis informed key principles, which were ranked by a Consumer Reference Group and an Expert Reference Group to define a framework for digital health promotion strategies supporting self‐management of chronic disease risk. These informed the development of a “Quick Guide” for digital health promotion to support risk management in women. The guide was refined through iterative feedback and tested for acceptability by health promotion professionals (*n* = 6).

**Results:**

Key themes included the need for tailored and accessible health information, convenience and affordability, and the recognition of how fear and mistrust influence health behaviours. Community connection and storytelling were prominent, as was targeting younger audiences through education and social media. The Quick Guide was deemed acceptable by professionals, especially useful for early‐career practitioners.

**Conclusion:**

This co‐design project led to a practical Quick Guide to support the development of community‐driven digital health promotion strategies for managing chronic disease risk in healthy women.

**So What?:**

Future research may explore its real‐world application in digital campaigns and test its integration into primary care and community health services.

## 1 Introduction

Chronic diseases are the leading causes of illness, disability, and mortality among Australian women. In 2020–2021, 56% of Australian females reported having one or more chronic conditions, highlighting the significant burden these diseases impose on women's health [1]. Among these, 40% can be prevented by managing risk factors [2]. There are risk assessment tools that estimate individual risk of chronic disease [3, 4, 5] but their use has not been reported. Screening programs are effective in reducing mortality through early disease detection; however, participation rates consistently fall below recommended targets [6].

In Australia, consumers are defined as people with lived experience of a health issue, including patients, carers, relatives, and people who represent the health interest of a specific community group [7]. There is growing evidence that a co‐design approach involving consumers in the development of health interventions may improve the uptake of strategies to manage chronic disease risk. For example, Anderson et al. applied co‐design methodologies to identify practical strategies and develop interventions to improve participation in Australia's National Bowel Cancer Screening Program [8]. In another study, community health workers underwent a two‐day training program and co‐designed and co‐facilitated culturally tailored cervical screening forums to improve participation in cervical screening in culturally and linguistically diverse (CALD) migrant and refugee women in Western Sydney, Australia [9]. Co‐design methodologies have also shown promise in improving chronic disease risk management among Indigenous Australian women. Dissanayake et al. conducted a single‐arm pre–post‐trial to evaluate the *Hope for Health program—*a co‐designed dietary and lifestyle intervention developed in partnership with senior Yolŋu women from a remote Aboriginal community in northeast Arnhem Land [10].

These studies demonstrate the potential of co‐design methodologies to address persistent barriers to chronic disease prevention and early detection among women in Australia. Whether through improving screening uptake, tailoring health promotion to the needs of culturally diverse groups, or embedding interventions within Indigenous knowledge systems, co‐design offers a flexible and responsive approach to improving engagement. By centring consumer perspectives and adopting a co‐design approach in the development of health promotion strategies, it may be possible to achieve measurable gains in screening and risk assessment participation to support chronic disease risk management.

However, while co‐design has shown promise in improving engagement with screening, significant gaps remain. Existing co‐design studies have focused on single screening programs or specific population groups and do not address how women understand and manage chronic disease risk across multiple conditions. Little is known about how asymptomatic women engage with online risk assessment tools or what features would support preventive behaviours in a digital context. There is currently no co‐designed framework that integrates women's perspectives across chronic disease areas to guide the development of practical health promotion tools.

To address this gap, this study used a co‐design process to identify women's priorities, barriers, and preferences for managing chronic disease risk and translated these insights into a digital health promotion framework. The aim was to generate a user‐informed Quick Guide to support practitioners developing digital preventive health strategies for women in Australia.

## 2 Methods

**FIGURE 1:**
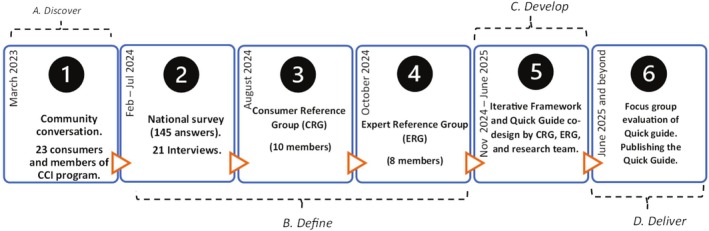
Double diamond research framework aligned with the project's timeline.

A double diamond co‐design framework [11] was used, given its effectiveness when addressing processes that require problem exploration and iterative refinement. Using the four phases of the framework—discover, define, develop, and deliver—we explored ways to improve the prevention and early detection of chronic diseases—specifically bowel, breast, and cervical cancers, as well as diabetes and cardiovascular disease—among asymptomatic women in Australia. Each phase informed the sequencing of research activities, stakeholder engagement, and the iterative co‐design process (See Figure 1).

### 2.1 Discover

This study targeted asymptomatic women (including people assigned female at birth who are gender diverse) aged 18 years and over living in Australia. Although we did not set demographic quotas for specific communities, we aimed to involve as diverse a group of women as possible across all stages of the project. To support this, we used open recruitment through community networks, existing partnerships and word‐of‐mouth. This naturally facilitated participation from women with varied lived experiences, including those from metropolitan, regional and rural areas, as well as migrant, LGBTQIA+, and Aboriginal and Torres Strait Islander communities. This approach enabled broad representation without directing recruitment toward any particular subgroup.

The initial phase of our study (Figure 1, Stage 1) involved an online community conversation in March 2023, in collaboration with Western Australia's Consumer and Community Involvement (CCI) Consumer Program. Promotional materials, including flyers and social media posts, were disseminated through the programs' consumer networks and affiliated health and community organisations across Facebook, Instagram, LinkedIn and Twitter. Women interested in participating were invited to register their attendance in advance. Twenty‐eight women from across Australia attended the online session, 16 consumer (or community members), 7 members of the Consumer and Community Involvement program and 5 research team members. They represented a broad age range (18–65 years), with most aged 18–43 years and were predominantly located in Western Australia (68.8%), followed by New South Wales (18%), Victoria (6.3%) and South Australia (6.3%). Attendees reported diverse cultural backgrounds, including Australian, Asian, European, and Aboriginal and Torres Strait Islander identities.

The online conversation was conducted in four breakout rooms, with the consumer program staff serving as facilitators and researchers acting as scribes. This informal open dialogue aimed to explore strategies for enhancing the prevention and early detection of chronic diseases specific to women in Australia. This approach generated valuable insights and a wide range of ideas from women with lived experience and/or interest in chronic disease and preventive health. Additionally, it offered participants a supportive and inclusive environment to reflect on their individual experiences and actively contribute to the conversation. Participants shared their self‐perceived risks of chronic diseases, along with their knowledge and awareness of online risk assessment tools and screening programs that target diabetes, cardiovascular disease, breast, cervical and bowel cancer. A key idea that emerged was the concept of “one‐stop shop” (web‐based resource)—a centralised, easily accessible platform that consolidates comprehensive information and resources specifically for women to support their risk management of multiple chronic diseases.

### 2.2 Define

#### 2.2.1 Survey and Interviews

As part of the “Define” phase (Figure 1, Stages 2, 3 and 4), the WeManage Project, “Women Encouraging the Management of Chronic Disease Risk*”* project was established to build upon the Community Conversation. The project started with a non‐representative anonymous online survey between February and July 2024 to assess women's use and attitudes toward existing screening programs and risk assessment tools. The survey questionnaire was developed and refined collaboratively by the research team, which included two consumer co‐lead investigators. Specifically, the survey was reviewed for cultural appropriateness by three members of the research team who held established community leadership roles and contributed diverse lived‐experience perspectives relevant to women's health and health service engagement. These perspectives included experience in breast cancer advocacy, LGBTQIA+ community engagement, and migrant and humanitarian community development work. Attention was given to how questions were framed, especially around prevention and risk communication, to ensure they were accessible and respectful to participants from broad diverse backgrounds.

Survey questions captured participants' awareness and use of screening for breast, cervical and bowel cancer, and online risk assessment tools for diabetes (AUSDRISK) [3], breast cancer (iPrevent [5]) and cardiovascular disease (Australian CVD Risk Calculator [4]). The survey also gathered information on respondents' health practices and demographics. Survey respondents were recruited through snowball sampling via social media, community networks, and health sector stakeholders with targeted efforts to reach women across metropolitan, regional, and rural areas across Australia. At the conclusion of the survey, respondents were invited to indicate their willingness to participate in a follow‐up one‐on‐one interview.

Interview questions (*n* = 9) were developed collaboratively by the investigator team. The questions explored participants' perceptions of health and chronic disease risk, access to relevant health information, and barriers to engaging in preventive health behaviours. Participants were also invited to suggest ways to increase uptake of screening and online risk assessment tools. Additionally, the interviews sought feedback on the proposed web‐based resource, including suggestions regarding its content, usability, and promotional strategies.

#### 2.2.2 Consumer and Expert Reference Groups

Concurrently, a Consumer Reference Group (CRG) was established to ensure inclusion of women with diverse lived experiences and geographic and cultural backgrounds. Formed in August 2024, the group consisted of 11 women aged between 18 and 74 years, including eight members residing in WA, two in Victoria, and one in Queensland. Members were selected after submission of an expression of interest and subsequent interviews. Each member was actively engaged in the women's health sector, representing diverse roles such as researchers, health sciences students, community leaders or advocates, or consumers with lived experience of chronic disease. One member of the CRG currently serves as a co‐lead of the research team. The Expert Reference Group (ERG) was established in October 2024 and included eight professionals from across Australia with specialised knowledge in cardiology, endocrinology, breast and cervical cancers, as well as cancer screening and risk assessment.

#### 2.2.3 Analyses

A descriptive analysis of survey data was conducted using SPSS v.27, and an initial thematic analysis of the interview data was carried out using NVivo. Interview data were analysed using an inductive, reflexive analysis following Braun and Clarke's six‐phase approach [12, 13, 14]. The first author read each transcript and undertook line‐by‐line coding generating initial codes directly from the data. Coding was iterative, with codes refined throughout the process as new insights emerged. A second researcher independently reviewed a subset of transcripts to assess the alignment of the initial coding. The first author and second researcher met to discuss and refine the preliminary code list. The first author then collated codes into initial thematic categories, which were reviewed and further refined through discussion within the broader investigator team. Through this collaborative process, the initial categories were developed into 10 thematic principles. These principles were subsequently ranked during an online group workshop involving both the CRG and the investigator team. The ranked principles were then presented to ERG for further evaluation and refinement in a follow‐up discussion, ensuring a comprehensive, multi‐stakeholder perspective in the development of the web‐based resource framework. Based on the outcomes of these discussions and ongoing deliberation within the research team, the thematic principles were refined into a consolidated draft framework comprising five core principles.

### 2.3 Develop

The combined, iterative feedback from consumers on the framework enabled the research team to redefine the project's focus, leading to an organic outcome: the development of a “Quick Guide” to inform digital health promotion strategies, rather than the originally proposed web‐based resource (“one‐stop‐shop”) discussed during the community conversation. The proposed purpose of the Quick Guide was to support stakeholders, including grassroots health promotion professionals, when developing digital health promotion strategies. The Quick Guide summarised the framework's principles and translated them into a practical checklist. It was then circulated to both the CRG and ERG for further feedback and validation.

### 2.4 Deliver

A national online focus group was conducted with six health promotion professionals to assess the acceptability of the Quick Guide. The session was informed by the Theoretical Framework of Acceptability (TFA) of healthcare interventions developed by Sekhon et al. [15]. Four of the seven TFA constructs were selected as most relevant: appeal, clarity, effectiveness, and usability. These constructs were adapted into questions and prompts to guide the focus group discussion. The session explored participants' answers to each question, and their responses were analysed thematically. Based on this feedback, the Quick Guide was further refined to incorporate their recommendations.

## 3 Results

### 3.1 Survey

Sociodemographic characteristics of survey respondents (*N* = 145) are presented in Table 1. Respondents were predominantly aged between 18 and 44 years (63%) and living in urban areas (89%). Most of them (74%) were residing in Western Australia. Although most respondents (60%) were born in Australia, 40% were born overseas. Among respondents born outside of Australia, 6.2% were born in Africa, 5.5% in Asia or the Middle East, 3.4% in Latin America, 2.8% in Europe, and 2.1% in North America (results not shown in Table 1). This proportion is higher than the estimated national average of overseas‐born Australian residents, which stands at 31.5% [16]. Survey respondents were highly educated, with over half (60%) having completed a bachelor's degree.

**TABLE 1:** Sociodemographic characteristics of survey participants and interviewees.

Variables	Survey participants (*N* = 145)			Interviewees (*N* = 21)		
	Valid	Missing	(%)	Valid	Missing	(%)
Age						
18–24			22			19
25–39			24			19
40–44	145	0	17	21	0	19
45–54			17			10
55+			20			33
State of residence						
WA			74			47
VIC			8			24
QLD			7			14
SA	126	19	5	21	0	5
NSW			3			5
ACT			1			0
NT			1			5
TAS			1			0
Remoteness						
Urban			89			
Rural, regional or Remote	112	19	11			
Country of birth						
Australia			60			
Other			27			
England	131	14	5			
India			5			
New Zealand			2			
Italy			1			
Education						
Post‐grad			34			
Bachelors			26			
Diploma/TAFE	129	16	23			
Year 12 and under			17			

*Note:* Empty cells indicate variables for which data were not available in the respective sample.

**FIGURE 2:**
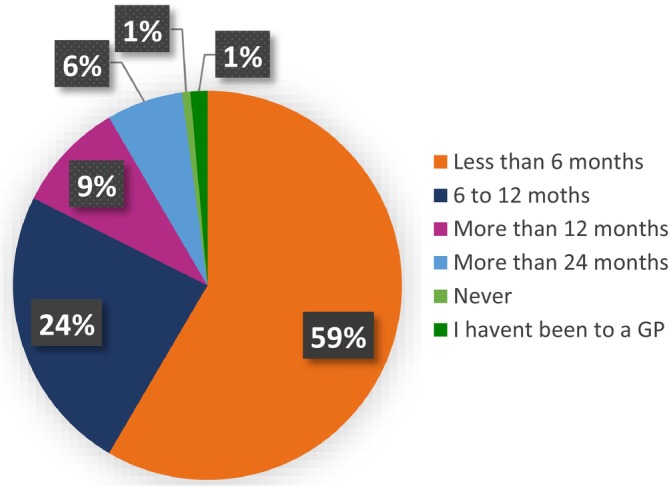
Time since last general health discussion with a General Practitioner (GP).

In terms of health practices, 83% reported consulting their GP at least once a year, and more than half had done so within the past 6 months (Figure 2). More than 70% of eligible survey respondents reported both awareness and uptake of recommended screening tests (Table 2). Eligibility varied by screening program: 49.7% of respondents were age‐eligible for mammography, 76.6% for cervical screening, 29.7% for FOBT, and 23.4% for the AUSDRISK tool. Specifically, 93% were aware of their eligibility for mammograms based on age outlined in Australian national guidelines, and 78.6% had undergone at least one; 94.3% were aware of their eligibility for cervical screening and 91.2% had participated; and 92.5% were aware of their eligibility for the Faecal Occult Blood Test (FOBT), with 75% having completed at least one. These figures are based on the subset of respondents eligible for each screening test. In contrast, awareness and uptake of the diabetes screening tool (AUSDRISK) were markedly lower. Over half of the eligible respondents (60.5%) were unaware of their eligibility, and approximately 60% reported never having completed the AUSDRISK tool (Table 2).

**TABLE 2:** Eligibility awareness and uptake of screening tests among eligible women.

	Mammogram	Cervical	FOBT	AUSDRISK
	(*n* = 72)	(*n* = 111)	(*n* = 43)	(*n* = 34)
Eligibility awareness				
Yes	93	94.3	92.5	39.5
No or unsure	7	5.6	7.5	60.5
Uptake				
Had at least one	78.6	91.2	75	41.3
Haven't had any/unsure	21.4	8.8	25	58.8

*Note:* Values are expressed as percentages.

Most survey respondents had not used online risk assessment tools (70%, Table 3) and had not undergone genetic testing to assess their risk of chronic disease (84%, Table 3).

**TABLE 3:** Self‐reported use of chronic disease risk management tools among survey respondents (*N* = 145).

Variables	Valid	Missing	(%)
Risk assessment tools			
Yes			23
No	136	9	70
I don't know			7
Genetic testing			
Yes			13
No	134	11	84
I don't know			3

### 3.2 Interviews

**FIGURE 3:**
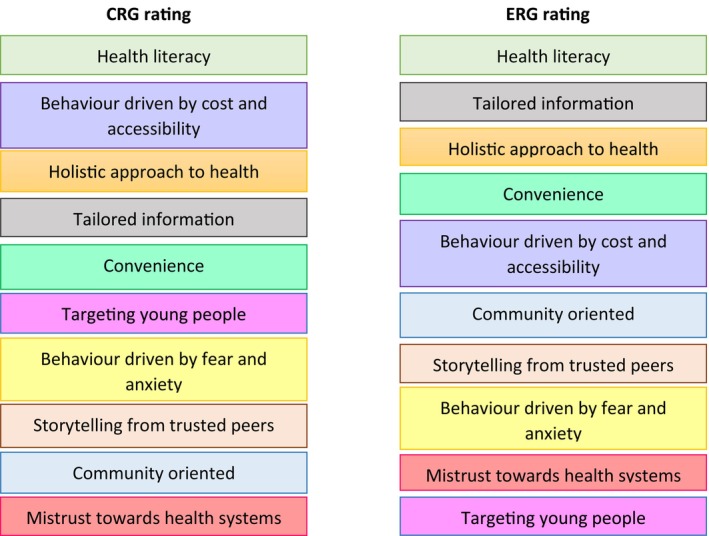
Comparison of the ratings of thematic principles between members of the Consumer Reference Group (CRG) and Expert Reference Group (ERG) members.

Of the 21 interview participants, nearly 60% were aged between 18 and 44 years, with 81% born in Australia. The majority resided in Western Australia (47%) and Victoria (24%) (Table 1). The 10 thematic principles presented were developed through analysis of these interviews. The principles were subsequently ranked in a participatory workshop with CRG members and later discussed and ranked separately by ERG members. CRG and ERG members rated the thematic principles in broadly similar ways, with alignment on some key areas (Figure 3). Both groups ranked health literacy, tailored information, holistic approach to health, and convenience among the five top priorities, indicating shared emphasis on clear communication, personalised messaging, and accessible health care. They also agreed on the lower priority of mistrust toward health systems and of behaviour driven by fear and anxiety.

The primary differences in rankings between the two groups related to the emphasis placed on community themes, specifically, community‐oriented and storytelling from trusted peers and the theme of targeting young people. CRG members ranked community‐oriented themes relatively lower, while the ERG positioned them in the middle tier. In contrast, CRG members placed greater emphasis on targeting younger audiences than ERG members.

### 3.3 Core Principles

**FIGURE 4:**
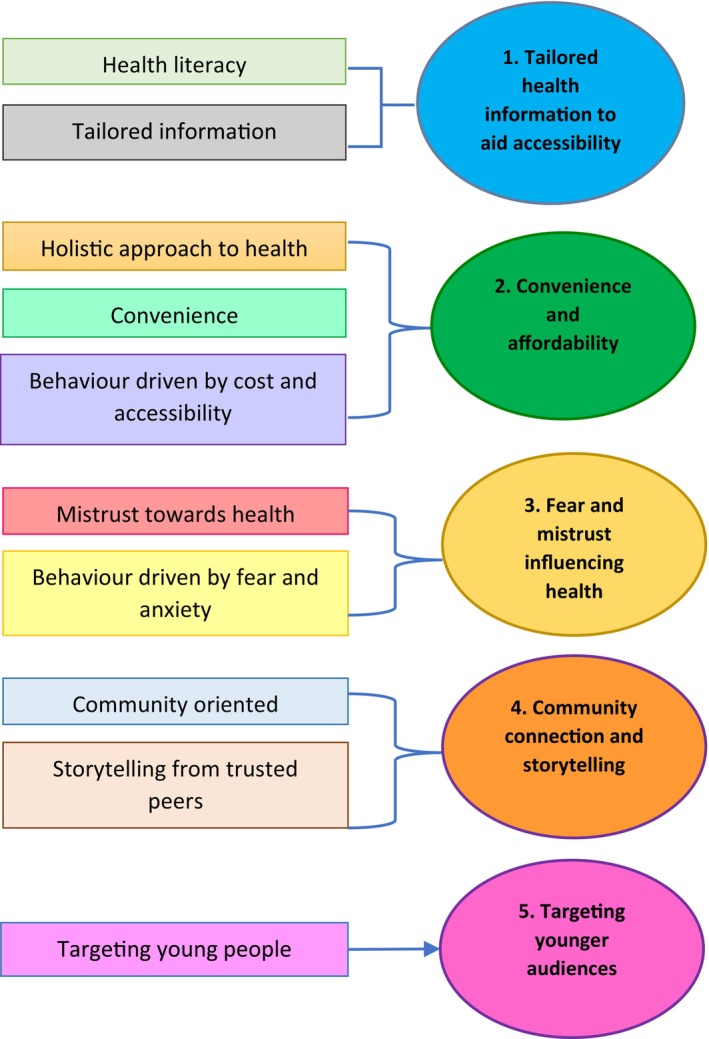
Recategorization of thematic principles into five core principles.

The 10 thematic principles were consolidated into core principles (Figure 4) following discussions among research team members, and the core principles are described below.

#### 3.3.1 Tailored Health Information to Aid Accessibility

There was a strong consensus among research participants, as well as members of the consumer and expert reference groups, that health information within any health promotion strategy must be tailored to the health literacy levels of the intended audience. Participants frequently found that existing health information is often overly technical and complex, rendering it difficult to understand and inaccessible to individuals without a background in health. One participant highlighted, *“*The only people that would really use it [the web‐based resource] are people who are educated and interested in their health, and it's the people that we're not targeting.” (*Health promotion practitioner in the multicultural sector*). Another added, “For someone who's not studying health, it [the web‐based resource] can be very daunting.” (*Health sciences student*).

Participants recommended that content be organised in a straightforward and accessible manner, based on the specific health needs of the user. They emphasised the importance of presenting information in manageable sections to avoid overwhelming users, rather than displaying all information at once. An interviewee suggested, “Filter out information that people might not need at the time they're visiting the website.” (*Women's health professional*).

Additionally, participants advocated for a holistic approach to health information that integrates nutrition, social environment, and overall health. They emphasised the need to frame mental health and reproductive issues, such as Polycystic Ovary Syndrome (PCOS) and endometriosis, as chronic diseases, highlighting their connections to other chronic diseases to promote a more integrated understanding of health. One participant observed, *“*I think about all the aspects that make up my health and well‐being—physical, mental, emotional, occupational, financial, environmental…acknowledging that these are dependent on each other; they can't be siloed.” (*Disability advocate*) Another noted, “Mental health issues impact female bodies more in terms of hormonal regulation.” (*Health sciences student*).

#### 3.3.2 Convenience and Affordability

Participants emphasised that digital health promotion strategies should be convenient and easy to access to attract users, particularly given the wide availability of social media content (e.g., health channels on Instagram, TikTok). One participant remarked, “[A web‐based resource] has to be so unique, relatable, personable…there's so many things by so many organisations” (*Health promotion practitioner in the multicultural sector*), another suggested, “Link it into anything that's already existing in that space.” (*Disability advocate*).

The health resources promoted on any health website should also be convenient as some interviewees noted difficulties in accessing health services due to long wait times and high costs. Two participants shared, “We have to wait at least two months to get a test done at scanning centres” (*Participant living in a regional area*) and “A lot of people our age [18–25] are reluctant to see a GP because it's really expensive right now.” (*Health sciences student*).

#### 3.3.3 Fear and Mistrust Influencing Health Behaviours

Health behaviours among some women are influenced by fear. In some cases, fear motivates a proactive approach toward disease prevention and health management. One participant noted, “If you had statistics that were…confronting…people you're targeting might see that and realise, oh, I'm actually at risk” (*Health promotion practitioner in the multicultural sector*). However, fear can also have the opposite effect, discouraging women from seeking care or following up on their health. One respondent mentioned, “My sister never checked her breasts…she didn't want to go to the doctor…she said, ‘I don't want that confrontation’.” (*Lived experience advocate*).

Mistrust in health systems can also impact engagement with health promotion strategies and follow‐through on GP advice. One participant expressed doubts, “With an online assessment tool, I'm not sure how people will trust that and follow up on the results.” (*Lived experience advocate*). Another shared, “I don't think doctors are as switched on as they used to be.” (*Health promotion practitioner in the multicultural sector*).

#### 3.3.4 Community Connection and Storytelling

A sense of community is highly valued among participants and plays a critical role in health promotion. Within digital health strategies, this may involve features fostering community connections and linking users to local health services. For example, one interviewee suggested, “A sub‐option [within the web‐based resource] to help people connect, so they feel supported and motivated to use the website.*”* (*Health sciences student*). Another recommended, “[Include] resources where it [the web‐based resource] shows bulk billing clinics.” (*Health sciences student*).

Storytelling through community champions and relatable narratives was also viewed as essential for promoting tool usage, particularly for culturally and linguistically diverse (CALD) communities. One participant remarked, “[Have] people that go out into the community, who are like them, talk about it [the web‐based resource].” (*Lived experience advocate*). Another participant noted the need for a hands‐on approach, especially for multicultural women: “They [multicultural women] would use it [any web‐based resource] if it's in a group setting…where we guide them through it together.” (*Health promotion practitioner in the multicultural sector*).

Two participants stressed the importance of relatable stories: “[Include] stories that are heartfelt, interesting, personal, relatable,” (*Health promotion practitioner in the multicultural sector*) and “If people can see how that [health promotion tool] relates to them, whether that person is from their culture, or maybe they're a mother, or someone who was healthy themselves.” (*Lived experience advocate*).

#### 3.3.5 Targeting Younger Audiences

Participants identified the need to target younger women to raise awareness and encourage the use of health promotion tools early in life, ideally through schools. They felt that early education could normalise preventive health practices. One participant observed, “I definitely think increasing education toward younger girls in school…puts the idea in their head that these things aren't just for older people.” (*Health sciences student*).

During discussions with researchers and reference group members, participants noted that promoting these tools could also positively influence the health behaviours of the mothers and grandmothers of the younger generation.

Social media was also recognised as an effective way to reach younger audiences. One young participant shared, “For my age group [18–25], that's [social media] the main source of information…I've never seen any ads on my Instagram about it [screening tests and risk assessment tools]. But if I did see an ad, I'd be more inclined to go and research it.” (*Health sciences student*).

### 3.4 Focus Group

The two‐page Quick Guide can be found in Supporting Information, developed based on the five core principles and includes a checklist framed as a series of questions to be used when designing or promoting digital health promotion strategies for women. Results of the national online focus group, involving five health promotion professionals, categorised acceptability by appeal, clarity, effectiveness, and user‐friendliness.

#### 3.4.1 Appeal

Participants consistently described the Quick Guide as visually and structurally appealing due to its concise, two‐page format. They appreciated its alignment with public health priorities and its practical focus, describing it as a “useful resource” and “appealing for professionals” due to its brevity and relevance. However, some participants recommended enhancing the appeal further by including links to examples of successful programs or strategies.

#### 3.4.2 Clarity

The guide was generally seen as clear and easy to interpret, especially for those with a background in health promotion. Its step‐by‐step format and checklist structure were described as accessible and straightforward. One participant noted it would be “a great resource for a new graduate in health promotion planning.” However, the absence of a specific prompt regarding program evaluation was flagged as a limitation, suggesting that clearer guidance on assessing impact would enhance its utility.

#### 3.4.3 Effectiveness

Participants felt the Quick Guide was likely to be effective in supporting health promotion planning, particularly because it was co‐designed with consumers and grounded in evidence‐based practice. They emphasised that the guide addressed user needs by using plain language and offering actionable strategies, such as leveraging digital media. Nonetheless, they cautioned that perceived effectiveness would depend on uptake, promotion, and sustained updates to maintain relevance. Inclusion of budgeting prompts was also suggested to improve practical planning outcomes.

#### 3.4.4 User‐Friendliness

All participants reported feeling confident using or sharing the guide in professional contexts. It was described as “an action plan” and “a good reminder document” that supports structured thinking. Participants indicated it was suitable for use in tertiary education, professional development, and practice‐based settings. Some concerns were raised about the need for background knowledge to fully utilise the guide and the importance of contextual adaptation to meet local needs.

## 4 Discussion

This study demonstrates the value of a consumer‐led, co‐design approach in developing digital health promotion strategies for chronic disease risk management among asymptomatic women. Using the double diamond framework, we integrated survey findings, community member interviews, and iterative engagement with both a Consumer Reference Group (CRG) and an Expert Reference Group (ERG) to develop and evaluate a two‐page Quick Guide. The findings highlight both alignment and divergence in stakeholder priorities, reveal gaps in awareness of risk assessment tools, and reinforce the need for strategies that embed accessibility, cultural relevance, and community‐centred communication.

### 4.1 Gaps in Awareness and Use of Preventive Tools

Despite high awareness and reported uptake of screening programs such as mammography, cervical screening, and faecal occult blood tests, supported by national campaigns and eligibility‐based invitations, there were clear gaps in awareness and use of personalised risk assessment tools. Most survey respondents were unaware of online risk assessment tools or when they are eligible to use them. These findings reflect limitations in current outreach and suggest a need to move beyond general health promotion.

Women in this study viewed chronic disease risk holistically, integrating multiple areas of health rather than treating conditions as isolated categories. Evidence from other Australian settings similarly demonstrates that some women prefer holistic and relationship‐centred models of care. For example, First Nations women's engagement with cervical screening improves when prevention discussions occur within trusted GP relationships that address health in a broader, integrated manner [17]. This suggests that efforts to increase awareness and use of risk‐assessment tools may be more effective when integrated into women‐centred models of care that recognise the broader context of women's lives. In this context, digital health promotion strategies may need to integrate multiple resources or adopt cross‐promotional approaches that align with how women organise and act on health information.

### 4.2 Divergent Priorities Between CRG and ERG Members

The comparison of rankings between CRG and ERG members offers insight into differing perspectives between community members and professionals. While both groups valued health literacy and accessibility, CRG participants placed relatively greater emphasis on targeting younger people, describing them as both future health consumers and current contributors of intergenerational health knowledge given that they can influence the health behaviours of older family members. This was particularly evident among women from migrant backgrounds where multigenerational and reciprocal care are arrangements common. These findings align with evidence showing that, within many Australian multicultural families, younger women may act as informal conduits of health knowledge, shaping how older relatives access, interpret, and act upon health information [18]. This supports the view that chronic disease risk management strategies may be strengthened by recognising intergenerational roles within households.

In addition, CRG members prioritised individual‐level concerns impacting their health behaviours (i.e., cost and accessibility of health services), in contrast, ERG members placed greater emphasis on peer‐led storytelling and community‐oriented approaches. These differences may reflect variations in lived experience versus health promotion design priorities. The inclusion of both groups throughout the design process helped identifying and integrating both individual‐level and structural considerations into the final Quick Guide.

### 4.3 Strengths and Limitations of the Co‐Design Process

The co‐design process produced a practical, community‐informed Quick Guide that health promotion professionals viewed as clear, useful, and actionable. The use of a structured framework, combined with multiple rounds of feedback and iterative refinement, enhanced the tool's relevance and acceptability.

A key strength was the project's capacity to adapt responsively to stakeholder input. Feedback from both CRG and ERG members supported a strategic shift from developing a centralised digital platform to co‐producing a Quick Guide. This type of adaptation is characteristic of authentic co‐design, which emphasises responsiveness to emerging insights and iterative refinement [19]. Co‐design literature, including experience‐based co‐design, shows that modifying project goals and outputs as participant perspectives evolve strengthens alignment with end‐user needs [19]. Our pivot reflects these principles, although it also required changes to research activities, evaluation tools, and dissemination plans, which may limit comparability with other studies.

The online format supported participation across broader geographic regions but may have reduced accessibility for participants with lower digital literacy. Future work could address these limitations by including region‐specific workshops or in‐language sessions.

### 4.4 Acceptability and Implementation Considerations

Evaluation of the Quick Guide using the Theoretical Framework of Acceptability (TFA) indicated strong affective and ethical alignment, particularly in terms of its co‐designed origins, clarity, and plain‐language checklist format. Health promotion professionals reported confidence in using or recommending the tool, particularly for student training or as a planning prompt. However, some concerns were noted: absence of explicit evaluation guidance, need for strategic linkage to community examples, and challenges in adapting the guide for diverse local settings.

These findings are consistent with implementation research demonstrating that the acceptability and effective use of health‐promotion tools depend not only on their clarity and relevance but also on the presence of practical guidance and integration within existing service structures [20] The Consolidated Framework for Implementation Research (CFIR) identifies clear instructions, adaptability, and organisational support as central determinants of successful adoption [20]. The broader literature similarly emphasises that interventions and resources are more likely to be used when end‐users understand how to apply them in practice and when training, examples, and contextual supports are available [21]. This aligns with participant feedback on the Quick Guide, particularly regarding the need for evaluation guidance, community‐specific examples, and dissemination through existing professional development channels.

### 4.5 Implications for Future Health Promotion Strategies

This study emphasises the importance of designing health promotion strategies that align with women's lived realities, particularly in the context of asymptomatic chronic disease risk. Tools must be both culturally and practically relevant, incorporating clear pathways for action, inclusive language, and a balance between individual responsibility and community‐level engagement. Engaging consumers in the design, ranking, and refinement processes, as done in the WeManage project, can help ensure that final products are not only acceptable but also sustainable and effective across contexts. Future research could explore the adaptation of the Quick Guide for specific population groups (e.g., regional, Aboriginal and Torres Strait Islander, or non‐English‐speaking women), evaluate its real‐world application in digital campaigns, and test its integration into primary care and community health services.

## Author Contributions

K.B., J.L.G.C., J.F., and J.S. conceptualised the study. P.B.‐A. carried out data collection, analyses, and wrote the initial draft of the manuscript. All authors revised the manuscript, approved the final manuscript as submitted, and agreed to be accountable for all aspects of the work.

## Funding

This project has been supported by the Women's Health Research, Translation and Impact Network, funded by the Australian Government's Medical Research Future Fund (Application ID EPCD000014, MRFF Emerging Priorities and Consumer Driven Research initiative). The research was also supported by a Cancer Australia, Priority‐driven Collaborative Cancer Research Grant (APP2012799).

## Ethics Statement

Ethics approval for this research was obtained from the University of Western Australia Human Ethics Research Committee 2023/ET000805.

## Consent

Informed consent was obtained electronically from all participants prior to participation.

## Conflicts of Interest

The authors declare no conflicts of interest.

## Supporting information


**File S1:** Quick Guide: Designing digital health promotion strategies for healthy women.

## Data Availability

The data that support the findings of this study are available on request from the corresponding author. The data are not publicly available due to privacy or ethical restrictions.
