# Supplementary material for: Community Co‐Design of a Quick Guide to Inform Digital Health Promotion Strategies for Asymptomatic Women at Risk of Chronic Disease
Source: Health Promot J Austr. 2026 Jul 26;37(4):e70222. doi: 10.1002/hpja.70222 (PMC13402771; doi:10.1002/hpja.70222)
Supplement: Supplementary file 1 — File S1: Quick Guide: Designing digital health promotion strategies for healthy women. [file HPJA-37-0-s001.pdf]

Quick Guide: Designing digital health promotion strategies for healthy women

Helping healthy women, those without symptoms, to manage their risk of chronic disease through accessible, engaging, online health promotion strategies.

As part of the WeManage project

What's the goal?

This quick guide is designed to help researchers, health promotion teams, and community leaders to create digital health promotion strategies that support women (including those recorded female at birth and gender diverse) in managing their risk of chronic disease. It has been co-designed with women, researchers, and health experts across Australia to make sure future strategies are relevant, easy to use, and effective.

Why it matters?

- Chronic diseases are the leading cause of death and disability in Australia, of which 40% can be prevented by managing risk factors [1].
- Screening programs effectively reduce mortality through early disease detection however, participation rates remain below recommended levels [2].
- Risk assessment tools that estimate individual risk are available [3, 4, 5], but their uptake has not been reported.
- A co-design approach helps develop trusted, user-friendly, and accessible health strategies that increase uptake and engagement [6, 7]

What we learned.

Funded by the Women's Health Research Translation Network, The WeManage Project (**W**omen **E**ncouraging the Risk **Manage**ment of Chronic Disease) is community-led and aims to research ways to improve the management of chronic disease risk in healthy women, those without symptom or disease. A national Community Conversation identified research priorities, leading to co-investigation of digital health promotion strategies for risk management. Guided by a Consumer Reference Group and informed by a national survey, interviews, and ongoing expert consultation, we identified what women want from an online health promotion strategy.

A digital health promotion strategy for women should:

Be simple and clear

- Use plain language. Avoid medical jargon.
- Present health holistically. Incorporate physical, mental, social and reproductive well-being.

Account for fear and mistrust

- Provide reliable sources and transparent information to build confidence.
- Clearly state how personal data will be managed and provide a straightforward process for women to remove their information if they choose to.

Reach younger women

- Introduce in schools and youth programs.
- Promote through social media.

Be convenient

- Require minimal clicks.
- Provide information tailored to users' specific needs (for example, interactive questions that filter out irrelevant content based on user responses).
- Address economic and time constraints (for example, provide links to local low-cost health service providers and availability).

Foster a sense of community

- Use local champions, peer support networks, and relatable storytelling to boost engagement . Women trust their peers more than institutions.
- Include a built-in chat and links to international forums or social media groups where women can connect over shared health concerns.

Health promotion strategy checklist:

Use these questions to ensure you're incorporating best practice at every stage.

User-centred and inclusive design	Build trust and engagement	Ensure sustainability
☐ Did you engage women at the start of your project?<br>☐ Is the strategy designed with accessibility in mind? (for example: easy navigation, clear instructions, text-to-speech, interactive visual content)<br>☐ Have you tailored content according to different demographic groups and user interests?<br>☐ Have you avoided medical jargon and used plain language?<br>☐ Did you consider cultural and linguistic diversity? (for example: translated content, visual aids)	☐ Are you working with community champions to promote the strategy?<br>☐ Does your strategy include real stories and testimonials from women?<br>☐ Are you promoting the strategy where women already seek information? (for example: social media, workplaces, schools, public spaces, women's health brands)<br>☐ Are you addressing common fears or mistrust about screening and health services?	☐ Does your strategy have a plan for regular updates and content refresh?<br>☐ Have you included ways for users to provide feedback and suggest improvements (for example: evaluation, analytics)<br>☐ Are you fostering a sense of community? (for example: discussion forums, peer support networks)<br>☐ Does the strategy remain relevant beyond the initial launch?

Final thought: Involve women at every step!

Women should be partners, not just users. Engaging them at every stage ensures a stronger sense of ownership and better quality outputs.

Need more help? For a more in-depth breakdown of women's insights, email us at WeManage-spgh@uwa.edu.au to request a copy of our publication regarding the co-design research process.

Feedback? If you would like to share your thoughts and/or experience using this Quick Guide, email us at WeManage-spgh@uwa.edu.au.

References

1. https://www.acdpa.org.au/
2. https://www.aihw.gov.au/reports/australias-health/cancer-screening-and-treatment
3. https://www.health.gov.au/resources/apps-and-tools/the-australian-type-2-diabetes-risk-assessment-tool-ausdrisk
4. https://www.cvdcheck.org.au/calculator
5. https://www.petermac.org/patients-and-carers/health-services-for-cancer-patients/cancer-prevention/iprevent
6. DeBortoli E, Soyer HP, Milne D, Dissanayaka N, Rae K, Gartner C, et al. Measurable outcomes of consumer engagement in health research: A scoping review. Frontiers in Public Health. 2022(10):994547.
7. Greenhalgh T, Hinton L, Finlay T, Macfarlane A, Fahy N, Clyde B, et al. Frameworks for supporting patient and public involvement in research: Systematic review and co-design pilot. Health Expectations. 2019; 22:785–801

Quick Guide Version 1.0 September 2nd 2025
